# Bacterial Abundance and Community Composition in Pond Water From Shrimp Aquaculture Systems With Different Stocking Densities

**DOI:** 10.3389/fmicb.2018.02457

**Published:** 2018-10-18

**Authors:** Yustian Rovi Alfiansah, Christiane Hassenrück, Andreas Kunzmann, Arief Taslihan, Jens Harder, Astrid Gärdes

**Affiliations:** ^1^Leibniz Centre for Tropical Marine Research (ZMT), Bremen, Germany; ^2^Laboratory of Marine Microbiology, Research Center for Oceanography, Indonesian Institute of Sciences, Jakarta, Indonesia; ^3^Balai Besar Pengembangan Budidaya Air Payau, Jepara, Indonesia; ^4^Department of Microbiology, Max Planck Institute for Marine Microbiology (MPI), Bremen, Germany

**Keywords:** *Litopenaeus vannamei*, Illumina sequencing, pathogenic bacteria, aggregates, salinity, Indonesia

## Abstract

In shrimp aquaculture, farming systems are carefully managed to avoid rearing failure due to stress, disease, or mass mortality, and to achieve optimum shrimp production. However, little is known about how shrimp farming systems affect biogeochemical parameters and bacterial communities in rearing water, whether high stocking densities (intensive system) will increase the abundance of pathogenic bacteria. In this study, we characterized bacterial communities in shrimp ponds with different population densities. Water quality, such as physical parameters, inorganic nutrient concentrations, and cultivable heterotrophic bacterial abundances, including potential pathogenic *Vibrio*, were determined in moderate density/semi-intensive (40 post-larvae m^-3^) and high density/intensive shrimp ponds (90 post-larvae m^-3^), over the shrimp cultivation time. Free-living and particle-attached bacterial communities were characterized by amplicon sequencing of the 16S rRNA gene. Suspended particulate matter (SPM), salinity, chlorophyll a, pH, and dissolved oxygen differed significantly between semi-intensive and intensive systems. These variations contrasted with the equal abundance of cultivable heterotrophic bacteria and inorganic nutrient concentrations. Bacterial communities were dominated by *Gammaproteobacteria, Alphaproteobacteria, Flavobacteriia, Bacilli*, and *Actinobacteria*. *Halomonas* and *Psychrobacter* were the most dominant genera in the particle-attached fractions, while *Salegentibacter, Sulfitobacter*, and *Halomonas* were found in the free-living fractions of both systems. Redundancy analysis indicated that among the observed environmental parameters, salinity was best suited to explain patterns in the composition of both free-living and particle-attached bacterial communities (*R*^2^: 15.32 and 12.81%, respectively), although a large fraction remained unexplained. Based on 16S rRNA gene sequences, aggregated particles from intensive ponds loaded a higher proportion of *Vibrio* than particles from semi-intensive ponds. In individual ponds, sequence proportions of *Vibrio* and *Halomonas* displayed an inverse relationship that coincided with changes in pH. Our observations suggest that high pH-values may suppress *Vibrio* populations and eventually pathogenic *Vibrio*. Our study showed that high-density shrimp ponds had a higher prevalence of *Vibrio*, increased amounts of SPM, and higher phytoplankton abundances. To avoid rearing failure, these parameters have to be managed carefully, for example by providing adequate feed, maintaining pH level, and removing organic matter deposits regularly.

## Introduction

*Litopenaeus vannamei* (Boone, 1931), white-leg shrimp, is an important species in aquaculture industry, which is widely reared in subtropical to tropical regions. They grow rapidly, have high survival rates even at high densities, possess a wide tolerance range of salinity and temperature, and can be cultivated in indoor (tanks or recirculating aquaculture systems) or outdoor facilities (ponds; Cuzon et al., 2004). Anderson et al. (2016) reported that world shrimp production in 2015 was 4.2 million tons of which *L*. *vannamei* contributed 75%. However, severe economic loss due to massive shrimp mortality has been occurring since 1987. For the period 1987–1994, economic loss reached USD 3 billion, representing 40% of the total production capacity of the industry (Brun et al., 2009; Walker and Winton, 2010). Moreover, a recent emerging bacterial disease, known as acute hepatopancreatic necrosis disease (AHPND), has occurred in Asian and Mexican shrimp aquaculture, causing an annual loss amounting to USD 1 billion with shrimp mortality exceeding 70% (Global Aquaculture Alliance [GAA], 2013, De Schryver et al., 2014; Soto-Rodriguez et al., 2015).

The intensification of the shrimp industries resulted in changes of farming systems and sustainability. Until the year 2000, 70% of shrimp farming in Indonesia was conducted in extensive pond systems (Kautsky et al., 2000), but recently changed to semi-intensive or intensive systems (Kementerian Kelautan dan Perikanan [KKP], 2015). Different shrimp stocking densities affect rearing processes, including a defined nutritional input and shrimp production. Robertson et al. (1993) reported that the shrimp growth rate increased progressively as feeding frequency increased from 1 to 4 times per day. However, only a portion of the nutrients in the feed is consumed, assimilated, and retained as shrimp biomass. Shrimps only incorporate 24 to 37% of nitrogen and 11 to 20% of phosphorus from the feed into their bodies (Funge-Smith and Briggs, 1998; Sahu et al., 2013). In addition, 15% of nitrogen losses occurs during the first 2 h of immersion of feed pellets into the pond water (Smith et al., 2002). These unused nutrients will lead to a change of pH and dissolved oxygen (DO) in the water column and pond sediment, eutrophication, proliferation of bacteria and plankton, and an increase of particulate organic matter (Avnimelech et al., 1994; Martin et al., 1998). Furthermore, Kautsky et al. (2000) reported that the risk of shrimp diseases often increased with culture intensity and high stocking densities. Thus, shrimp diseases, along with bad water quality management, might threaten shrimp farming sustainability.

Free-living (FL) and particle-associated (PA) bacterial communities from the same water sample can be distinct (Bidle and Fletcher, 1995; Riemann and Winding, 2001; Jain and Krishnan, 2017; Yang et al., 2017). As organic-rich particles, aggregates provide a suitable habitat for microorganisms to take up nutrients, and shelter from predators, as well as from destructive physical factors (Lyons et al., 2010; Kramer et al., 2013). In addition, aggregates can accommodate higher bacterial abundance and diversity than the adjacent water column (Kiørboe et al., 2003). Due to water movement in ponds, organic matter agglomerates and forms large flocs or aggregates (Hargreaves, 2013; Avnimelech, 2014), which might facilitate bacterial settlement and proliferation. Previous studies about bacterial community compositions (BCCs) in shrimp pond waters have yet to analyze FL and PA fractions separately (Sombatjinda et al., 2011; Zhang D. et al., 2014; Xiong et al., 2016; Hou et al., 2017), resulting in a paucity of information on BCC in both fractions.

Although the causative agents for bacterial diseases in shrimps have been identified (Lavilla-Pitogo et al., 1998; Kharisma and Manan, 2012; Soto-Rodriguez et al., 2015; Xiao et al., 2017), preventive efforts to minimize disease outbreaks seem to still be ineffective. Studies in temperate ecosystems showed that pathogenic bacteria have been found in aggregates (Lyons et al., 2005; Froelich et al., 2013). Based on that evidence, we propose that particle abundance can be used to estimate the potential proliferation of pathogenic bacteria in shrimp farming, and that controlling aggregates may become an effective tool to manage the spread and survival of pathogens. Therefore, it is necessary to investigate water quality parameters, bacterial abundance, and BCC from different shrimp farming systems for both FL and PA fractions, over shrimp cultivation time.

The current study aims to comprehend the effect of shrimp farming systems of different intensity on water quality parameters and BCC, to elucidate factors affecting bacterial communities, and to evaluate the presence of pathogenic bacteria, including pathogenic *V. parahaemolyticus* in the FL and the PA/aggregates fraction. We hypothesize that (i) different shrimp farming systems affect pond water quality, including suspended particulate matter (SPM) loading, bacterial abundance and community composition, and (ii) SPM loads more bacterial cells, including pathogenic bacteria.

## Materials and Methods

### Sample Collection and Sampling Sites

Water samples were collected between 9–10 a.m. from no water-exchange and plastic lining ponds (square shape in size 2700–3000 m^2^, water depth 1.3–1.5 m) of semi-intensive (40 post-larvae m^-3^, three ponds) and intensive (90 post-larvae m^-3^, three ponds) systems, during a cycle of shrimp rearing at day 10, 20, 30, 40, 50, 60, and 70 (September to November 2016). Shrimp ponds were located in Rembang Regency, Central Java, Indonesia (-6°37′41.13″ S 111°30′1″ E and -6°42′11.66″ S 111°21′54″ E, for semi-intensive and intensive system, respectively). Two liters of water were taken from 5 points of each pond at 1 m depth, and then mixed. Two liters of the mixed water were prepared for bacterial abundance and community analysis; 3 liters for SPM and inorganic nutrient analysis. Samples were stored in a cold and dark container, and transported to the laboratory at Diponegoro University, Semarang, Indonesia, for immediate analysis. Remaining water was used for physical parameter measurement. Total harvest of each pond was recorded at the end of shrimp rearing.

### Environmental Parameters

Salinity, temperature, pH, chlorophyll a, DO, and turbidity were measured *ex situ*, using calibrated Manta Eureka 2 multi-probes (Eureka Environmental Engineering, TX, United States).

Of each water sample, 0.3 L were filtered through a 0.45 μm syringe filter and the filtrate was poisoned with 1.2 mL of a 0.35 g L^-1^ HgCl_2_ solution for inorganic nutrient analysis (ammonium: NH_4_^+^, nitrate: NO_3_^-^, nitrite: NO_2_, phosphate: PO_4_^3-^ and silicate: SiO_4_^4-^). The samples were stored at -20°C until analysis. Inorganic nutrient measurements were done in triplicates at the Laboratory of Chemistry, Research Center for Oceanography (LIPI), Jakarta, Indonesia, according to the colorimetry method by Strickland and Parsons (1972), with a Shimadzu UV-1800 spectrophotometer.

Suspended particulate matter was measured as dry mass on pre-combusted GF/F filters (porosity 0.7 μm, ø 47 mm, VWR, France) in triplicates after filtration of a known volume of water sample (0.1–0.5 L). Weight of the filters was determined using a precision balance (ME 36S, Sartorius, Göttingen, Germany) after drying the filters for 24 h at 40°C.

### Bacterial Abundance

To obtain cultivable heterotrophic bacterial number, 100 μL of water samples (dilution factor 10^-2^–10^-5^) were plated onto Marine Agar 2216 (Difco, United States) and incubated at 28°C (room temperature) for 48 h. While potentially pathogenic *Vibrio* were isolated by inoculating 100 μL of undiluted to 10^-4^ diluted water sample onto selective Thiosulfate Citrate Bile Salts Sucrose(TCBS) medium (Roth, Karlsruhe, Germany), followed by incubation at 35°C for 24 h.

Samples for total bacterial cell counts were prepared by fixing 50 mL water with 4% v/v paraformaldehyde and stored at 4°C for 24 h. The dilution factors for the samples were as follows: undiluted for day 10 samples, 5 × 10^-1^ for day 20 and 30 samples, 10^-1^ for day 40, 50, and 60 samples, and 5 × 10^-2^ for day 70 samples. Ten milliliters of diluted fixed samples were subsequently filtered through 3.0 and 0.2 μm polycarbonate filters (ø 47 mm and 25 mm, respectively, Whatman, Dassel, Germany) to determine bacterial cells in the particle-attached (PA) and the free-living (FL) fractions, respectively. For the FL fractions at day 30, 40, and 70, only 5 ml filtrates were filtered. Filters were air dried and stored at -20°C for further staining. A 4′,6-diamidino-2-phenylindole (DAPI) staining was performed according to Kepner and Pratt (1994) for selected samples (10, 40, 50, 60, and 70 days samples). Filters were stained with 1 μg mL^-1^ of DAPI solution for 5 min, then washed in 80% ethanol and rinsed with sterile distillated water. Stained filters were air dried in the dark for 30 min, and then mounted with 10 μL of mounting solution consisting of 3:1 Citifluor AF mounting medium (Citifluor Ltd., London, United Kingdom) and Vectashield (Vector Laboratories Inc., Burlingame, United States). Bacterial cells, as well as size and number of aggregates were observed under a fluorescence microscope *Axio Imager.D2* (Zeiss, Jena, Germany) at 1000× magnification. Bacterial cell abundance in FL filters was calculated from 30 photos per filter, using the free software *ImageJ*. Bacterial cells in PA filters were manually counted from 10 aggregates per filter of similar size. Sizes of aggregates were determined using a net micrometer grid (12.5 mm × 12.5 mm, divided into 10 × 10 fields, which is equal to 15,625 μm^2^ at 1000× magnification). The cell average per filter was then divided by volume of filtered samples multiplied by dilution and factor of effective filter area (which was 31888 at magnification 1000×, for a filtration funnel with a diameter 25 mm, Millipore, Darmstadt, Germany) and number of aggregates (4 aggregate sizes per filter, **Supplementary Table 1**), for the FL and the PA fraction, respectively.

### Molecular Analysis of Bacterial Communities

Five hundred milliliters of water samples were filtered subsequently through 3.0 and 0.2 μm polycarbonate filters (ø 47 mm, Whatman, Dassel, Germany) for PA and FL bacterial fractions, respectively. Genomic DNA was extracted according to Nercessian et al. (2005). DNA pellets were dissolved in TE buffer (10 mM Tris-HCl, 1 mM EDTA, pH 8.5). DNA concentrations were measured photometrically and checked for purity (ratio of light absorption at 260–280 nm) using a nanoquant plate reader (Infinite M200 Pro, Tecan, Germany). 16S rRNA gene amplification was performed from genomic DNA extracts from days 10, 40, 50, 60, and 70, considering cultivable bacterial abundance information (heterotrophic bacteria and potential pathogenic *Vibrio*) and bacterial disease evidence (white feces disease), which occurred at previous rearing cycles between 50 and 65 days of rearing (personal communication with shrimp farm owners).

DNA sequences of the V3–V4 hypervariable region of the 16S rRNA gene were obtained from amplicon sequencing with the primer set S-D-Bact-0314-b-S-17 (5′-CCTACGGGNGGCWGCAG-3′)/S-D-Bact-0785-a-A-21 (5′-GACTACHVGGGTATCTAAKCC-3′; Klindworth et al., 2013). Sequencing at LGC genomics (Berlin) was done on an Illumina MiSeq using the V3 Chemistry (Illumina) in a 2 × 300 bp paired-end run. Demultiplexing, i.e., grouping of sequences by sample, and the removal of the primer sequences from the raw paired-end reads were performed by LGC genomics (Berlin, Germany). Further bioinformatic analysis steps were carried out at ZMT, Bremen, Germany, according to Hassenrück et al. (2016). Sequences were quality trimmed with a sliding window of 4 bases and a minimum average quality of 15 with *trimmomatic* v.033 (Bolger et al., 2014). Quality trimmed sequences were merged using PEAR v0.9.8 (Zhang J. et al., 2014). The swarming approach was used to cluster OTUs using *swarm* v2.1.1 (Mahé et al., 2014). For each OTU, one representative sequence (seed sequence) was taxonomically classified with SINA (SILVA Incremental Aligner) v1.2.11 using the SILVA rRNA project reference database (release 128) at a minimum alignment similarity and quality of 0.9 and a last common ancestor consensus of 0.7 (Pruesse et al., 2012). Unwanted lineages (such as archaea, chloroplasts, and mitochondria), as well as singletons and doubletons, i.e., OTUs occurring only once or twice in the data set, were removed. Rarefaction curves were calculated based on OTU richness and inverse Simpson index to assess the quality of sequence data sets. Samples with fewer than 500 sequences were excluded from the dataset for the analysis.

DNA sequence datasets were deposited in the European Nucleotide Archive (ENA) with the project accession number PRJEB26390, using the data brokerage service of the German Federation for Biological Data (GFBio, Diepenbroek et al., 2014), while biogeochemical data were archived in PANGAEA^1^.

### Detection of Virulence Genes via PCR

Detection of toxin genes, i.e., transcriptional regulator (*toxR*), thermolabile haemolysin (*tlh*), thermostable direct haemolysin (*tdh*), *Photorhabdus* insect-related (*pirA* and *pirB*) was performed in a Mastercycler^®^ (Eppendorf, Nexus gradient, Hamburg, Germany) using the set of primers described in **Supplementary Table 2**. As these genes are common genes in several representative of the genus *Vibrio* (i.e., *V. cholera, V. vulnificus, V. parahaemolyticus, V. alginolyticus, and V. owensii*), we designed specific primer pairs which only amplify DNA sequences belonging to *V. parahaemolyticus* as this bacterium causes most of bacterial diseases in shrimp (Soto-Rodriguez et al., 2015; Heenatigala and Fernando, 2016; Xiao et al., 2017). Ten samples, which had a high amplicon abundance of *Vibrio*, were chosen for PCR analysis (**Supplementary Table 3**). The PCR conditions were as follows: a reaction mixture consisted of 2 μL (20 ng μL^-1^) of template, 2 μL of 10× PCR buffer B containing 15 mM MgCl_2_, 0.5 μL of 25 mM MgCl_2_, 0.5 μL of 0.2 mM dNTPs, 1 μL of 0.1 mM forward and reverse primer, 0.1 μL of 5U μL^-1^ Taq polymerase (all reagents provided by Roboklon EURx, Berlin, Germany) and 12.9 μL sterile distillated water. The amplification conditions were as follows: pre-denaturation at 95°C for 3 min, followed by 40 amplification cycles consisting of denaturation at 95°C for 30 s, annealing at 60°C for 15 s, and extension at 72°C for 30 s, and a final elongation at 72°C for 5 min. *Vibrio parahaemolyticus* DSM 11058 (DSMZ, Braunschweig, Germany) was used as positive control for those toxin genes, while *Vibrio vulnificus* DSM 10143 (DSMZ, Braunschweig, Germany) served as negative control.

### Data Analysis

To examine differences in environmental parameters and bacterial abundances between culturing intensity (intensive and semi-intensive systems), and among days, as well as the interaction between culturing system and sampling day, general linear mixed models (GLMM) were performed with shrimp pond as random factor. Data were log-transformed to achieve normal distribution prior to statistical testing. *Tukey’s post hoc* tests were applied in cases where there were significant differences among sampling days and/or an interaction between sampling day and shrimp farming system. Forward model selection based on the Akaike information criterion (AIC), considering collinearity of the variables and variance inflation values, was used to determine environmental parameters, which best explained observed counts of cultivable heterotrophic bacteria and potential pathogenic *Vibrio*. Total shrimp harvest was tested with one-way ANOVA and total bacterial cell numbers from DAPI counting of FL and PA bacterial communities were analyzed using MANOVA. As first-level *post hoc* test, the effect of significant predictor variable (here: sampling day) in MANOVA of FL and PA bacterial numbers was then tested with individual GLMMs per fraction. Furthermore, pairwise comparisons between sampling days were conducted as second-level *post hoc* test. Repeated measures correlation between FL and PA bacterial cell numbers was estimated using rmcorr R package (Bakdash and Marusich, 2018).

Principal component analysis (PCA) was conducted to examine the relationship among environmental parameters, as well as cultivable bacterial abundances, and to characterize shrimp ponds of the different farming systems over time. Bray–Curtis dissimilarity coefficients were calculated to investigate variation of OTU compositions in FL and PA communities among ponds, as well as within and between the systems. Patterns of BCC in both fractions was visualized by non-metric multidimensional scaling (NMDS) based on Bray-Curtis dissimilarities using the function *metaMDS* of the *vegan* R package (Oksanen, 2017). Analysis of similarity (ANOSIM) was performed to test for differences in community composition in free-living and particle-attached fraction between systems over the time.

Redundancy analysis (RDA) was performed to explore relationships between bacterial communities and environmental variables in FL and PA fractions, for OTUs occurring in at least 10 samples, and with a sequence proportion of at least 1% in one sample. Data sets were separated by size fractions, and then centered log-ratio-transformed using *aldex.clr* (Fernandes et al., 2014) before being tested. Forward model selection based on the AIC, followed by collinearity and variance inflation inspection, was used to select the environmental parameters best suited to explain patterns in BCC. The significance of the individual parameters of the RDA models was assessed using restricted permutation tests to account for the repeated measurements within ponds. For the PA fraction, data from day 60 was excluded due to a missing value to ensure equal numbers of observation from each pond.

Population turnover, which occurred in the FL and PA fractions, was estimated for certain interesting OTUs of the genera *Salegentibacter, Psychrobacter, Halomonas*, and *Vibrio*, using the fraction of read abundance times cells method (FRAxC) proposed by Kevorkian et al. (2018), where bacterial cell numbers in the FL and PA of each selected sampling day were multiplied with the relative sequence proportion of 16S rRNA genes. We assumed that the biases associated with sampling procedure, DNA extraction, amplification, and cell counts did not vary over time. FRAxC results were also used to estimate correlation between estimated *Vibrio* abundance and selected environmental parameters (SPM, salinity, pH, temperature, ammonium, phosphate, and nitrite).

All statistical analyses, as well as figure visualizations were performed in R (R version 3.4.2, R Core Team, 2017, using R Studio v.0.98.1056) with the packages vegan (Oksanen, 2017), nlme (Pinheiro et al., 2017), ALDEx2 (Fernandes et al., 2014), rmcorr (Bakdash and Marusich, 2018), and gplots (Warnes et al., 2016).

## Results

### Shrimp Culturing Conditions

Total shrimp harvest differed significantly between semi-intensive and intensive systems (one-way ANOVA, *F*_1,4_ = 111.43, *p* < 0.01). The intensive system showed a twofold higher production than semi-intensive system, which were 3,950 ± 284 kg and 1,990 ± 151 kg, for intensive and semi-intensive final harvest (mean ± SD, *n* = 3 per system). There was a high variability among the ponds within each system for turbidity, chlorophyll a, pH, salinity, DO, and inorganic nutrients during rearing time, which increased over time (**Table 1**). Physical parameters such as salinity, pH, DO, chlorophyll a, and SPM were significantly different between the two systems (GLMM, *p* < 0.05, **Supplementary Table 4**). Among sampling days, we detected differences in temperature, salinity, pH, turbidity, chlorophyll a, SPM, cultivable heterotrophic bacteria and potential pathogenic *Vibrio* (GLMM, *p* < 0.05, **Supplementary Table 4**). The interaction between day and farming system was also significant for temperature, salinity, and turbidity. However, there were no significant differences between semi-intensive and intensive systems for dissolved inorganic nutrients, temperature, and turbidity (GLMM, *p* < 0.05, **Supplementary Table 4**).

**Table 1:** Physical parameters, in-organic nutrient concentrations, and cultivable heterotrophic bacteria abundances over 70 rearing days in the semi-intensive and intensive farming system.

Day	10		20		30		40		50		60		70	
System^a^	S	T	S	T	S	T	S	T	S	T	S	T	S	T
Parameters^b^														
SPM^∗^ (mg L^-1^)	66.6 ± 8.0^a^	68.7 ± 5.2^a^	68.1 ± 4.9^a^	70.3 ± 7.7^a^	80.1 ± 12.2^b^	91.5 ± 8.3^b^	96.5 ± 5.7^c^	122.0 ± 22.2^c^	158.1 ± 4.0^d^	202.6 ± 18.5^d^	167.3 ± 5.5^d^	187.6 ± 11.0^d^	173.1 ± 2.2^d^	171.3 ± 17.7^d^
Chl a**^∗^** (mg L^-1^)	13.34 ± 7.13^ac^	5.47 ± 3.82^a^	14.61 ± 1.49^ae^	10.11 ± 5.04^ab^	11.22 ± 0.96^ab^	22.8 ± 19.8^ae^	18.5 ± 5.5^ae^	77.9 ± 32.1^e^	20.7 ± 9.1^ae^	63.5 ± 29.1^ce^	30.9 ± 32.3^ae^	33.7 ± 14.2^bce^	16.9 ± 14.9^acd^	68.1 ± 27.7^de^
pH**^∗^**	8.24 ± 0.04^b^	8.18 ± 0.11^b^	8.20 ± 0.14^ab^	7.95 ± 0.07^ab^	8.26 ± 0.22^ab^	7.9 ± 0.1^ab^	8.5 ± 0.1^b^	8.0 ± 0.1^b^	8.3 ± 0.2^ab^	7.6 ± 0.5^ab^	7.9 ± 0.1^a^	7.8 ± 0.1^a^	8.4 ± 0.5^b^	8.1 ± 0.1^b^
DO**^∗^** (mg L^-1^)	6.13 ± 0.14	6.32 ± 0.1	6.4 ± 0.02	6.36 ± 0.13	5.90 ± 0.65	6.2 ± 0.1	6.1 ± 0.2	6.9 ± 0.8	5.9 ± 0.3	6.1 ± 0.2	5.7 ± 0.1	6.3 ± 0.2	5.8 ± 0.5	6.2 ± 0.3
Salinity**^∗^** (PSU)	35.37 ± 0.15^bd^	38.43 ± 0.78^d^	35.20 ± 0.69^ad^	36.79 ± 0.50^cd^	32.44 ± 3.26^a^	35.9 ± 0.9^bd^	33.7 ± 0.7^abc^	35.0 ± 0.9^abc^	33.4 ± 0.3^ab^	35.8 ± 0.3^bd^	33.6 ± 1.4^abc^	34.1 ± 0.8^abc^	34.6 ± 0.9^abc^	33.6 ± 0.5^ab^
Temperature (°C)	31.24 ± 0.41^cd^	30.19 ± 0.50^bcd^	28.08 ± 0.64^a^	30.44 ± 0.18^bcd^	31.61 ± 0.98^d^	30.4 ± 1.6^bcd^	29.6 ± 0.2^ac^	28.9 ± 0.5^ab^	31.0 ± 0.1^cd^	30.1 ± 0.4^bcd^	30.8 ± 0.8^cd^	30.2 ± 0.1^bcd^	30.7 ± 0.5^bcd^	30.5 ± 0.4^bcd^
Turbidity (NTU)	16.53 ± 10.69^ac^	3.9 ± 2.21^a^	24.5 ± 8.67^bc^	9.93 ± 8.44^ab^	35.87 ± 30.26^bc^	22.9 ± 14.6^bc^	27.0 ± 10.5^bc^	25.7 ± 2.7^bc^	25.9 ± 12.9^bc^	25.3 ± 5.6^bc^	27.6 ± 7.2^bc^	30.2 ± 14.4^c^	26.9 ± 9.2^bc^	35.7 ± 15.9^c^
Cultivable Bact. (log10 CFU mL^-1^)														
THB	4.75 ± 0.06^ac^	4.42 ± 0.15^a^	5.35 ± 0.04^bcd^	4.65 ± 0.30^ab^	6.29 ± 0.15^ef^	5.50 ± 0.16^cd^	5.99 ± 0.50^de^	6.76 ± 0.20^eg^	6.87 ± 0.69^fg^	7.44 ± 0.19^g^	7.53 ± 0.19^g^	7.41 ± 0.36^g^	7.47 ± 0.18^g^	7.4 ± 0.1^g^
TPPV	2.03 ± 0.56^a^	2.13 ± 0.90^a^	4.35 ± 0.36^bc^	3.32 ± 0.32^ab^	4.51 ± 0.19^bc^	3.98 ± 0.18^b^	3.85 ± 0.29^b^	3.73 ± 0.41^b^	3.85 ± 0.48^b^	3.72 ± 0.15^b^	4.16 ± 0.69^bc^	5.41 ± 0.89^c^	4.18 ± 0.42^bc^	4.36 ± 0.7^bc^
Nutrient (mg L^-1^)														
NH_4_^+^	0.60 ± 0.37	0.436 ± 0.671	0.46 ± 0.71	0.15 ± 0.08	0.31 ± 0.23	0.26 ± 0.34	0.23 ± 0.19	0.26 ± 0.22	0.50 ± 0.07	0.37 ± 0.30	0.61 ± 0.858	0.835 ± 0.248	0.33 ± 0.34	0.36 ± 0.25
NO_2_^-^	0.004 ± 0.004	0.183 ± 0.292	0.004 ± 0.004	0.008 ± 0.007	0.021 ± 0.025	0.005 ± 0.003	0.007 ± 0.007	0.201 ± 0.334	0.016 ± 0.006	0.001 ± 0.001	0.002 ± 0.001	0.079 ± 0.113	0.013 ± 0.009	0.004 ± 0.003
NO_3_^-^	0.048 ± 0.06	0.075 ± 0.11	0.007 ± 0.005	0.097 ± 0.16	0.004 ± 0.003	0.027 ± 0.042	0.028 ± 0.02	0.213 ± 0.184	0.034 ± 0.025	0.014 ± 0.013	0.052 ± 0.04	0.307 ± 0.456	0.028 ± 0.026	0.284 ± 0.484
PO_4_^3-^	0.33 ± 0.31	0.53 ± 0.41	0.29 ± 0.20	0.50 ± 0.37	0.22 ± 0.17	0.22 ± 0.27	0.31 ± 0.38	0.72 ± 0.07	0.39 ± 0.26	0.11 ± 0.12	0.35 ± 0.36	0.59 ± 0.47	0.61 ± 0.08	0.31 ± 0.22
SiO_4_^4-^	1.424 ± 1.453	0.206 ± 0.191	0.179 ± 0.047	0.315 ± 0.253	1.217 ± 0.918	0.343 ± 0.134	0.565 ± 0.576	0.324 ± 0.245	0.898 ± 1.193	0.45 ± 0.36	0.636 ± 0.792	0.11 ± 0.07	0.623 ± 0.668	1.157 ± 1.413

*Significant differences between systems at a significance threshold of 0.05 are indicated by asterisks; superscript letters indicate significant differences for the interaction system and day (p < 0.05). Data are shown as mean ± standard deviation. ^*a*^S, semi-intensive system; T, intensive system. ^*b*^SPM, suspended particulate matter; Chl a, chlorophyll a; THB, total heterotrophic bacteria; TPPV, total potential pathogenic bacteria.*

In both systems, pH decreased rapidly after day 40, but when limestones were added into the ponds, it increased from 7.92 ± 0.13 to 8.38 ± 0.53 and 7.63 ± 0.46 to 8.11 ± 0.13, in semi-intensive and in intensive system, respectively. Salinity decreased from 35.37 ± 0.15 to 32.44 ± 3.26 PSU and 38.43 ± 0.78 to 33.57 ± 0.45 PSU, in semi-intensive and in intensive system, respectively, due to freshwater input. Overall, the intensive system showed significantly higher salinities, SPM, and chlorophyll a concentration than the semi-intensive system, as well as higher variability of inorganic nutrients (**Table 1**).

The abundances of cultivable heterotrophic bacteria (THB) and potential pathogenic *Vibrio* (TPPV) in semi-intensive and intensive systems increased with rearing time, and peaked at day 60, at concentrations of 3.4 × 10^7^ CFU mL^-1^ of THB and 1.4 × 10^4^ CFU mL^-1^ of TPPV, and 2.6 × 10^7^ CFU mL^-1^ of THB and 2.65 × 10^5^ CFU mL^-1^ of TPPV, respectively (**Table 1**). Forward model selection showed that SPM, salinity, turbidity, temperature, and phosphate were major determinants for THB, while the determinants for TPPV were SPM, salinity, turbidity, pH, temperature, nitrite, and silicate.

**FIGURE 1:**
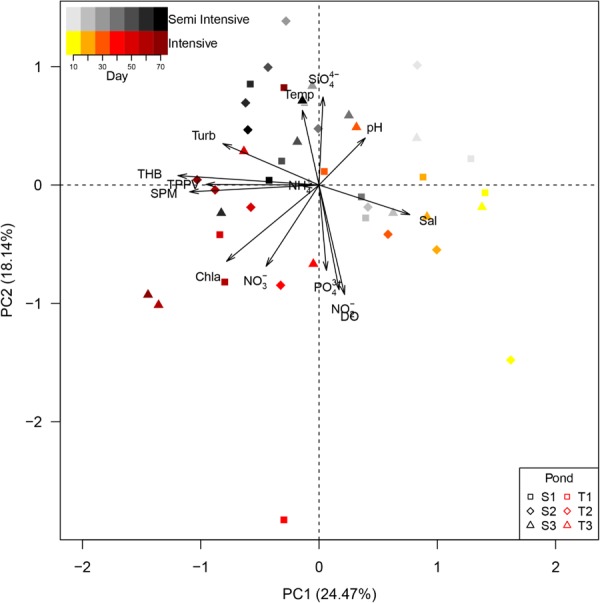
Principal Component Analysis of environmental parameters and bacterial abundances in intensive (T) and semi intensive (S) systems. Point shape indicates replicate pond of the same system. Increasing color intensity indicates rearing time. SPM, suspended particulate matter; THB, total heterotrophic bacteria; TPPV, total potential pathogenic *Vibrio*; NH_4_^+^, ammonium; Turb, turbidity; Temp, temperature; SiO_4_^4-^, silicate; Sal, salinity; PO_4_^3+^, phosphate; NO_2_^-^, nitrite; DO, dissolved oxygen; NO_3_^-^, nitrate; Chl a, chlorophyll a.

Principal component analysis for environmental parameters and cultivable bacterial abundances was able to retrieve 42.61% of the variation among ponds on the first two principal components (**Figure 1**). According to water parameters and cultivable bacterial abundances, there was a clear separation by shrimp farming systems based on rearing time. At the beginning of rearing (days 10 and 20), ponds of intensive and semi-intensive systems were clustered with high values of the first principal component, which was driven mainly by high salinity. Afterward, they were separated by the second principal component with ponds of the intensive system characterized by high concentrations of chlorophyll a. The abundances of THB and TPPV highly correlated to SPM (*Spearman* correlation, *rho*: 0.87 and *rho*: 0.40 for THB and TPPV, respectively).

Aggregates containing bacterial cells were found in different sizes. At day 10, small aggregates appeared which were formed by bacterial cells and their exudates. After 40 days, aggregates were composed of plankton, bacterial cells and bacterial exudates, causing the increase of aggregate sizes in both systems, as well as bacterial cell numbers. Aggregates of sizes between 937.5 and 1,406.5 μm^2^ were most abundant in both system over time, with the highest aggregate number at day 10 (511 ± 63 aggregates mL^-1^) and at day 40 (494 ± 27 aggregates mL^-1^), containing 86 ± 3 and 72 ± 6 bacterial cells per individual aggregate for semi-intensive and intensive pond waters, respectively (mean ± SD; *n* = 3; **Supplementary Figure 1** and **Supplementary Table 1**). Free-living (FL) bacterial cells of both systems increased and peaked at day 60 in concentration 3.8 × 10^7^ cells mL^-1^ and 5.0 × 10^7^ cells mL^-1^, for semi-intensive and intensive systems, respectively. Total particle-attached (PA) bacterial cell numbers were steady after day 40, with 6.8 × 10^5^ cells mL^-1^ and 6.4 × 10^5^ cells mL^-1^, for semi-intensive and intensive systems, respectively (**Table 2**). The FL cell numbers were positively correlated to the PA cell numbers (repeated measure correlation, *Spearman* correlation, rho = 0.50, *df* = 23, *p* = 0.01) and differed only among days (**Supplementary Tables 5, 6**).

**Table 2:** Total bacterial cell numbers in the free-living (FL) and the particle attached (PA) fractions.

Day	Fractions			
	FL [×10^7^ cells mL^-1^]		PA [×10^5^ cells mL^-1^]	
	S	T	S	T
10	0.06 ± 0.01^a^	0.09 ± 0.02^a^	0.62 ± 0.08^a^	0.50 ± 0.05^a^
40	1.83 ± 0.13^b^	3.09 ± 2.34^b^	6.77 ± 0.50^b^	6.40 ± 0.24^b^
50	2.94 ± 1.04^b^	2.13 ± 0.11^b^	6.51 ± 0.79^b^	6.55 ± 0.31^b^
60	3.79 ± 0.89^c^	5.01 ± 0.85^c^	6.16 ± 0.67^b^	6.09 ± 0.37^b^
70	3.47 ± 0.94^bc^	3.64 ± 1.22^bc^	7.68 ± 0.36^c^	7.42 ± 0.21^c^

*Data are shown as average ± standard deviation. Similar superscript letters after cell numbers in semi-intensive (S) and intensive (T) indicate no significant difference. Bacterial numbers in the FL and PA are interpreted separately. Complete statistical results were presented in **Supplementary Table 6**.*

### Bacterial Community Analysis

After removal of low-quality reads, 8,994–78,615 sequences, with on average 34,471 sequences per sample, were obtained from 59 samples. One sample was removed due to low sequence counts (<500 sequences). A total of 77,433 OTUs was obtained, ranging from 426 to 2465 OTUs, with an average of 1,312 OTUs per sample (**Supplementary Figure 2**).

**FIGURE 2:**
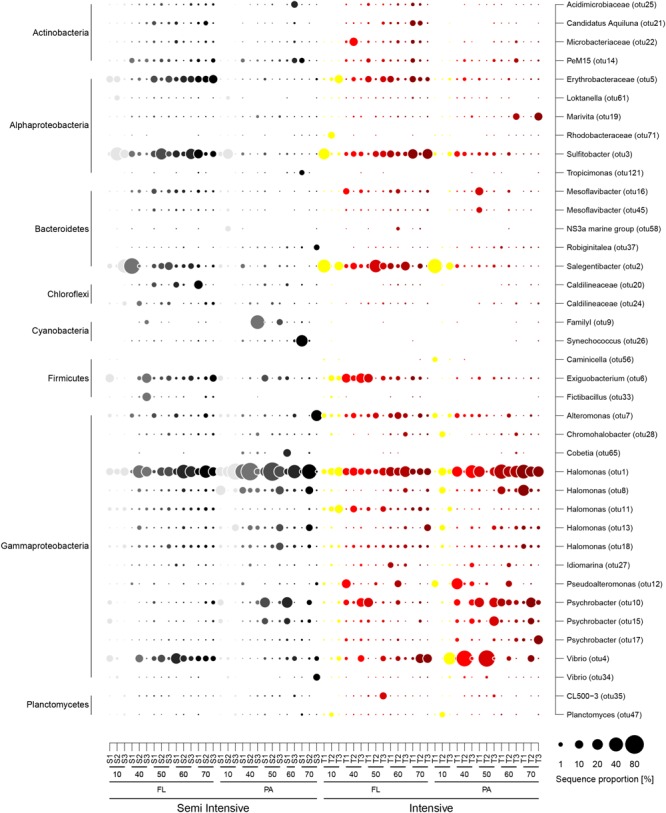
Contribution of the most abundant bacterial operational taxonomic units (OTUs) in the semi-intensive (light gray to black) and the intensive systems (yellow to dark red). Taxonomic affiliation for OTUs is provided for genus (Right) and class (Left) levels. S1–3 and T1–3: replicate pond for the semi-intensive and intensive ponds, respectively. Days of rearing are indicated below the pond symbols. FL, free-living fraction; PA, particle-attached fraction.

Most abundant bacterial OTUs in both systems belonged to the classes Acidimicrobiia, Actinobacteria, Alphaproteobacteria, Bacilli, Cyanobacteria, Flavobacteriia, and Gammaproteobacteria (**Figure 2**). Among them, the genera *Alteromonas, Erythrobacteraceae, Exiguobacterium, Halomonas, Vibrio, Pseudoalteromonas, Psychrobacter, Salegentibacter*, and *Sulfitobacter* were present in every sample. Cyanobacteria, such as Synechococcus and Cobetia, were frequently present in the semi-intensive system. The highest sequence proportion in the FL fraction of both systems belonged to Salegentibacter, Sulfitobacter, and Halomonas, while in the PA fraction *Psychrobacter, Vibrio*, and *Halomonas* were dominant, where the latter comprised up to 80% of the sequences in samples from the semi-intensive system after 40 and 50 days (**Figure 2**). In the SILVA reference database (SILVA 128 version), *Halomonas* OTU1 has a sequence similarity of 98 and 100% to *H. aquamarina* and *H. meridiana*, respectively.

Free-living and particle-associated bacterial communities from the same water sample were very different from each other as indicated by Bray–Curtis dissimilarity coefficients (**Table 3**). In addition, there was high variability in BCC within the same fraction among replicate ponds, which lead to a wide range in Bray-Curtis dissimilarity coefficients (0.27-0.97 and 0.33-0.83 for PA and FL fractions, respectively) between intensive and semi-intensive systems (**Table 4**).

**Table 3:** Bray–Curtis dissimilarities of bacterial community composition between free-living (FL) and particle-attached (PA) fractions.

Pond	Day				
	10	40	50	60	70
S1	0.54	0.83	0.83	0.85	0.91
S2	0.53	0.48	0.77	NA^∗^	0.54
S3	0.79	0.81	0.66	0.59	0.92
*Average*	*0.62*	*0.71*	*0.75*	*0.72*	*0.79*
T1	0.45	0.51	0.57	0.74	0.91
T2	0.68	0.87	0.91	0.49	0.69
T3	0.63	0.68	0.70	0.58	0.82
*Average*	*0.59*	*0.69*	*0.73*	*0.60*	*0.80*

*S, semi-intensive system; T, intensive; NA^∗^ indicated a missing sample for particle-attached fraction.*

**Table 4:** Bray–Curtis dissimilarities of bacterial community composition within system (among ponds) and between the systems for each fraction over the time.

Fraction	Day	System	Within system		Between system	
			Minimum	Maximum	Minimum	Maximum
FL	10	S	0.68	0.73	0.33	0.83
		T	0.66	0.85		
	40	S	0.62	0.92	0.63	0.84
		T	0.47	0.69		
	50	S	0.50	0.68	0.40	0.76
		T	0.58	0.83		
	60	S	0.41	0.60	0.41	0.69
		T	0.46	0.64		
	70	S	0.44	0.59	0.45	0.63
		T	0.55	0.66		
PA	10	S	0.69	0.71	0.70	0.98
		T	0.78	0.96		
	40	S	0.38	0.82	0.50	0.98
		T	0.52	0.91		
	50	S	0.62	0.76	0.43	0.99
		T	0.50	0.95		
	60	S	0.86	0.86	0.50	0.79
		T	0.50	0.65		
	70	S	0.94	0.97	0.27	0.97
		T	0.62	0.65		

*FL, free-living fraction; PA, particle-attached fraction; S, semi-intensive; T, intensive.*

**FIGURE 3:**
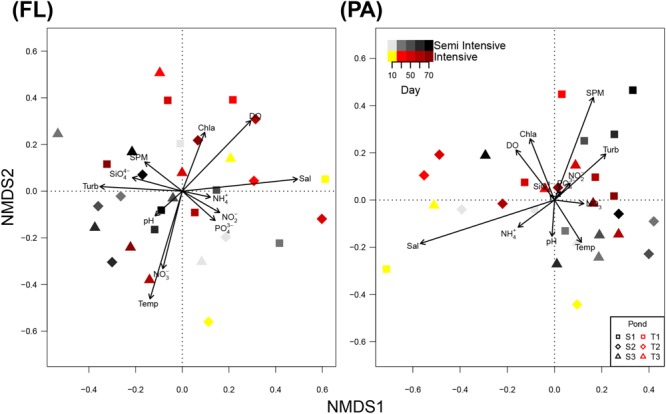
Non-metric multidimensional scaling (NMDS) plot of bacterial community composition (BCC) in the free-living (FL) and particle-attached (PA) fraction. Information on environmental parameters was added to the NMDS plot using *envfit.* Point shape indicates replicate ponds of the same system. Increasing color intensity indicates rearing time. SPM, suspended particulate matter; NH_4_^+^, ammonium; Turb, turbidity; Temp, temperature; SiO_4_^4-^, silicate; Sal, salinity; PO_4_^3+^, phosphate; NO_2_^-^, nitrite; DO, dissolved oxygen; NO_3_^-^, nitrate; Chl a, chlorophyll a.

NMDS showed a highly heterogeneous composition of the bacterial communities in the FL and the PA fraction for both systems (**Figure 3**). Analysis of similarity (ANOSIM) confirmed that there was no detectable pattern in FL or PA the BCC between system and day (**Supplementary Table 7**).

Redundancy analysis revealed that environmental variables explained 20.53 and 36.77% of the BCC in the FL and the PA fractions, respectively (**Supplementary Figure 3**). Among the observed environmental parameters, salinity was best suited to explain patterns in the composition of both FL and PA bacterial communities (*R*^2^ > 10%). Furthermore, chlorophyll a and nitrate had a minor effect on BCC in the FL fractions (**Table 5**).

**Table 5:** Contribution and significance of observe environmental factors which explain the variation in community composition of the free-living (FL) and the particle-attached (PA) fractions based on redundancy analysis (RDA).

Source of variation	Adjusted *R*^2^	*df*	*F*	*P*-value
FL				
Complete model (system + day)	0.103	5, 24	1.669	0.005
Sampling time (day)	0.070	4, 24	1.543	0.007
System	0.042	1, 24	2.172	0.204
Complete model (Sal + Chl a + NO_3_**^-^**)	0.153	3, 26	2.749	0.001
Salinity (Sal)	0.112	1, 26	4.558	0.001
Chlorophyll a (Chl a)	0.047	1, 26	2.510	0.005
Nitrate (NO_3_**^-^**)	0.030	1, 26	1.944	0.017
PA				
Complete model (system + day)	0.035	4, 19	1.207	0.184
Complete model (Sal)	0.128	1, 22	4.379	0.001

### Toxin Gene Assay, *Vibrio* Occurrence and Correlation to Environmental Parameters

**FIGURE 4:**
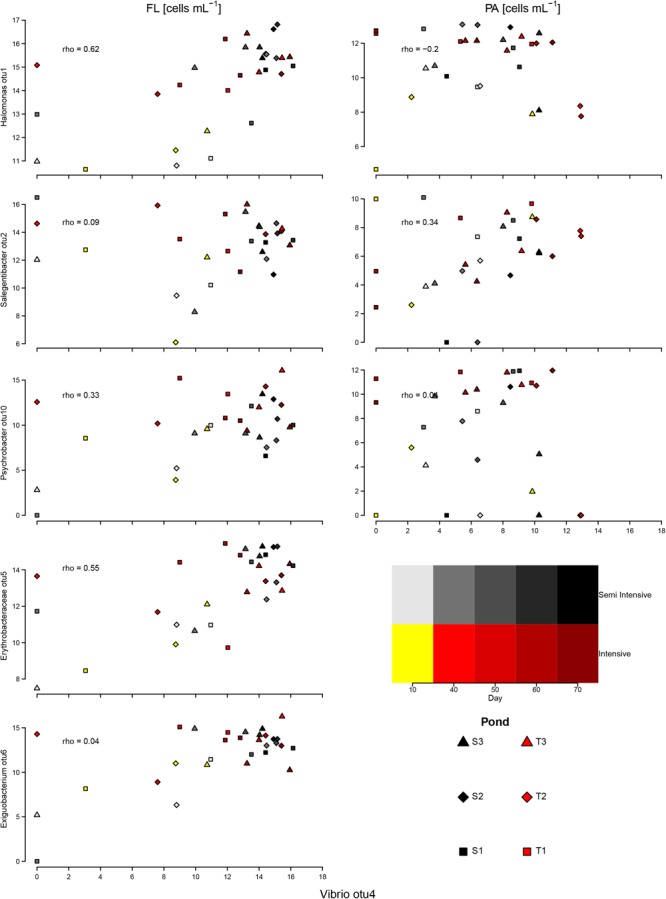
Correlation between estimated proportion of cells of the most-abundant OTUs in the FL and PA fractions and the dominant *Vibrio* OTU. The axes show log-transformed estimated bacterial numbers. rho, *Spearman* correlation coefficient.

All tested samples were negative for *toxR, tlh*, and *tdh*. These results indicate that no common pathogenic *V. parahaemolyticus* occurred in both systems, even though the given samples displayed high sequence proportions of *Vibrio*, specifically OTU4. The sequence of *Vibrio* OTU4 was identical to 16S gene sequences in the SILVA reference database belonging to *V. mytili, V. diabolicus*, and *V. parahaemolyticus*. The estimated abundance (FRAxC) of the dominant *Vibrio* OTU differed strikingly from that of the other dominant OTUs, such as *Halomonas, Psychrobacter*, and *Salegentibacter*, suggesting an inverse relationship. When FRAxCs of *Vibrio* were overly high, the proportions of the other genera were low. In the PA fraction, FRAxCs of *Vibrio* were negatively correlated to FRAxCs *Halomonas* (**Figure 4**).

FRAxCs of *Vibrio* in both fractions were positively correlated to SPM, temperature, ammonium, NP ratio, and TPPV, but they were negatively correlated to pH and salinity. In addition, *Vibrio* was negatively correlated to inorganic nitrite and phosphate in the FL fraction, although in the PA fraction the opposite trend was observed. In general, all correlation coefficients did not suggest strong correlation with values between -0.36 and 0.38 (**Supplementary Table 8**).

## Discussion

No water discharge shrimp aquaculture resulted in an accumulation of organic matter, through feed pellets, shrimp feces and other organic materials. Previous studies reported that degradation of organic matter, leaching of feed pellets, and release of ammonium from feces increase inorganic nutrients in the water column (Funge-Smith and Briggs, 1998; Smith et al., 2002; Burford and Lorenzen, 2004). These nutrients support bacterial and phytoplankton growth, which then leads to simultaneous change of physical parameters such as pH, DO, SPM, and turbidity (Burford and Williams, 2001; Burford et al., 2003).

In this study, shrimp pond water parameters and respective BCC in intensive and semi-intensive systems were compared. Contrary to our expectations, higher shrimp density in intensive farming systems did not significantly increase inorganic nutrients in the water column. We suggest that excessive nutrients were rapidly taken up by bacteria and phytoplankton as their abundance increased more strongly in the intensive system. This was also reflected in high SPM values in the intensive system due to the aggregation of phytoplankton cells. Phytoplankton can take up total ammonia nitrogen (TAN), while heterotrophic bacteria perform nitrification and nitrogen assimilation (Avnimelech, 2014). In pond water, photosynthesis by phytoplankton and cyanobacteria increases DO, while respiration and microbial activities, such as nitrification and sulfur oxidation decrease pH (Boyd and Tucker, 2014). The higher number of phytoplankton (which was indicated by Chl a) in intensive ponds might increase DO concentration. We supposed that photosynthesis might increase oxygen concentration. However, we are aware that our DO measurements only constitute daylight conditions and that due to the higher organic matter loading of the intensive system and in the absence of light, oxygen depletion via respiration processes may lower DO concentration below the levels of the semi-intensive system. Even though the intensive system resulted in higher abundances of phytoplankton and cultivable heterotrophic bacteria, we could show that the concentrations of harmful inorganic nutrients, such as ammonium and nitrite, DO, and pH were still far from lethal values for shrimps, as mentioned in other studies (Schuler, 2008; Avnimelech, 2014; Furtado et al., 2015).

We observed fluctuation of particle (aggregate or flocs) number over rearing time. We supposed that lower pellet input, sinking or consumption of flocs might cause the fluctuation of particle numbers. Water movement and exudate secreted either by bacteria or plankton lead to particle agglomeration (Grossart et al., 2006; Gärdes et al., 2011), generating bigger aggregates/flocs. When the aggregates sink to the bottom, particle numbers might decrease, resulting low turbidity/clearer water. Unfortunately, we did not measure daily feed input, sinking rate of aggregates and particle consumption by shrimp.

Environmental factors shape the structure and function of microbial communities (Allison and Martiny, 2008). Previous studies have shown that the succession of microbial communities was influenced by combinations of chlorophyll a, total nitrogen (TN), PO_4_^3-^, C/N ratio (Xiong et al., 2014), total phosphate, chemical oxygen demand (Zhang D. et al., 2014), and feed sources added into ponds (Qin et al., 2016). Of the parameters measured in this study, salinity was the most determinant variable which shaped BCC in the FL and particle-attached PA fractions, which concurred with previous studies (Yang et al., 2016; Hou et al., 2017; Kirchmann et al., 2017). We observed salinity fluctuation in both systems and supposed that rainfall and addition of sea water from reservoir pond to maintain rearing pond water level (∼140–150 cm) decreased salinity. During our study, rainfall could increase 10–20 cm of water level, while sea water was added more frequent in intensive ponds due to regular mud removal. However, it should be considered that the influences of salinity on the BCC in the FL fraction do not completely exclude the influence of other water parameters, such as unmeasured sulfur compound in water column like hydrogen sulfide (H_2_S) or sulfite which may explain the continually presence of *Sulfitobacter* in FL fraction.

Bacterial communities in intensive and semi-intensive systems were dominated by heterotrophic halophilic bacterial genera *Alteromonas, Erythrobacter, Halomonas, Pseudoalteromonas, Salegentibacter, Sulfitobacter*, and *Psychrobacter*. *Halomonas* and *Psychrobacter*, the salt-tolerant heterotrophic nitrifying bacteria (Chankaew et al., 2017), can oxidize ammonia in high concentration under saline conditions (Sangnoi et al., 2016). *Sulfitobacter* can oxidize sulfur and degrade high molecular weight dissolved organic matter (Bourne et al., 2004; Sosa et al., 2017). Other bacterial genera, such as *Salegentibacter, Exiguobacterium*, and *Erythrobacter*, were associated with shrimp shell degradation after molting (Sombatjinda et al., 2011). In our study, BCC differed from those reported by Sombatjinda et al. (2011) who analyzed *L. vannamei* earthen pond water with salinities close to freshwater. They identified *Nitrosomonas, Flavobacterium, Exiguobacterium, Synechococcus, Burkholderia, Nitrosospira*, and *Nitrobacter* as dominant bacteria (Sombatjinda et al., 2011). We assume that salinity caused the differences in BCC observed in our study, considering the salinity range of 32–38 PSU compared to 2–10 PSU in previous study (Sombatjinda et al., 2011). This is supported by a study of Chankaew et al. (2016), where common nitrifying bacteria, including *Nitrosomonas, Nitrosococcus, Nitrosolobus, Nitrospira, Nitrococcus*, and *Nitrobacter*, were absent in shrimp pond water with 20 PSU. Furthermore, the regular addition of commercial probiotics (personal communication with pond owners), which contained *Bacillus* sp., *Pseudomonas* sp, *Nitrosomonas* sp., *Aerobacter* sp., *Nitrobacter* sp., and *Nitrosococcus* sp. did not affect BCC in the FL and PA fractions of both systems, indicating that nitrification processes were generated by other microbes. We propose that in our study the dominance of particular heterotrophic halophilic bacteria may result in higher uptake of inorganic nutrient such as ammonium and nitrite (Sangnoi et al., 2016). Therefore, even in the intensive pond waters inorganic nutrients remained low. In our study, BCC changed over time, following a “resilience scenario” (Allison and Martiny, 2008), in which a replacement of bacterial taxa occurred due to environmental change, followed by a quick return to its pre-disturbance composition. This phenomenon can be clearly seen in the particle-attached fraction of the intensive system. When pH decreased below 8 at day 40 and 50, *Vibrio* replaced *Halomonas* as the most abundant OTU, but the sequence proportions of *Halomonas* recovered, after the pH increased at day 60 and 70. In contrast, BCC in the FL and the PA fractions of the semi-intensive system during the same period was similar while experiencing stable pH (above 8). We propose that pH was a disturbing factor for heterotrophic halophilic bacteria in our shrimp systems and lead to the change of BCC only in the intensive system. This is supported by Krause et al. (2012) who reported that slight changes in pH caused compositional shifts in marine bacterial communities.

*Vibrio*, a potential opportunistic pathogen for *L. vannamei* (Heenatigala and Fernando, 2016; Rungrassamee et al., 2016), has been found in higher proportions in the PA fractions of the intensive system. This might indicate that the particulate fraction, specifically marine aggregates, can accumulate potentially pathogenic bacteria, as suggested by other studies (Lyons et al., 2005; Froelich et al., 2013). Our hypothesis that the intensive system seemed to be more vulnerable to *Vibrio* outbreaks is supported by high abundance and recurrent presence of *Vibrio* spp. only in the intensive system. Therefore, it is necessary to maintain SPM and aggregate abundance and to avoid massive *Vibrio* growth due to the fact that *Vibrio* can convert from non-virulent to virulent under certain cell density threshold or if dramatic environmental changes occur (Zhou et al., 2012). Nevertheless, toxin genes (*toxR, tlh*, and *tdh*) of *V*. *parahaemolyticus* (Makino et al., 2003), as well as *pirAB* toxins for AHPND (Xiao et al., 2017) were not present in this study. We argue that other halophilic, potentially probiotic, bacteria might inhibit chromosome II replication, where those genes are located (Makino et al., 2003), as proposed by Defoirdt et al. (2011). As no other known probiotic taxa were detected in this study, we hypothesize that the presence of *Halomonas* in both systems might inhibit *Vibrio*. Recently, *Halomonas aquamarina* has been applied as probiotic in *L. vannamei* culture to oxidize ammonium and to prevent *Vibrio* growth, which then leads to an increase of survival rates of *L*. *vannamei* (Zhang et al., 2009; Suantika et al., 2013; Sangnoi et al., 2016). However, other unobserved microorganisms, such as bacteriophage, *Bdellovibrio, Saccharomyces, Streptococcus, Streptomyces*, and protists might also suppress *Vibrio* (Munro et al., 2003; Alagappan et al., 2010; Chavez-Dozal et al., 2013; Lakshmi et al., 2013; Kongrueng et al., 2017). Considering final shrimp harvests, the intensive system is the more economically promising system. However, shrimp pond management needs be improved to maintain water quality as well as beneficial bacterial communities. Regular feed control and mud discard were likely to have affected BCC in our study. Without regular mud discard, sludge and organic matter degradation might increase oxygen demand, and as a result oxygen might become depleted in the bottom of the shrimp pond. Under these conditions, anaerobic degradation of organic matter produces H_2_S, and increases other toxic inorganic compounds such as ammonia and nitrite. Furthermore, sludge deposit in bottom pond might enlarge the anaerobic area which may reduce the habitable space for the shrimp. While strictly adjusted feeding in semi-intensive system seemed to lead to a more stable BCC, regular mud discard in the intensive system, which was conducted once to twice per day, might have prevented the accumulation of toxic compounds (i.e., hydrogen sulfide, ammonium, and nitrite), as indicated by the lower proportion of *Sulfitobacter*. We suggest that giving adequate feed, discarding sludge, maintaining pH, and salinity, might avoid abrupt water quality change, eliminate large anaerobic areas at the pond bottom, and may minimize proliferation of potential pathogens, especially *Vibrio*. Some aspects of shrimp pond management related studies are given in Hopkins et al. (1993); Funge-Smith and Briggs (1998), and Avnimelech and Ritvo (2003). Consequently, it is necessary for pond aquaculture to provide sufficient seawater and sludge reservoirs to avoid environmental degradation risks due to sludge removal.

In view of BCC in FL and PA fraction over rearing period and the absence of virulence genes, we propose to apply “bioflocs” in shrimp aquaculture. This can be generated by adding carbohydrates such as molasses, rice bran or tapioca. Bioflocs might become an alternative food source for shrimps and be able to maintain ammonia and nitrite concentration in shrimp pond (Avnimelech, 2014). Moreover, because bacteria from commercial probiotics were not detected in any samples, we suggest that the regular application of these probiotics in such high salinity rearing pond is not necessary to be done. At the end, operational shrimp rearing costs, especially for feed pellet and commercial probiotics, can be reduced.

## Conclusion

Different stocking densities influenced water quality parameters, especially SPM, DO, chlorophyll a, and pH. High salinity shaped the BCC in the PA and FL fraction, favoring heterotrophic halophilic bacteria which facilitate an optimum uptake of inorganic nutrients and prevent increase of *Vibrio* growth. Despite a wide variability of BCCs in intensive and semi-intensive systems, the abundance of *Vibrio* was higher in the intensive system, which may therefore be more vulnerable to disease outbreaks. Monitoring BCC, especially the PA fraction’s, and larger particulates such as aggregates in shrimp pond waters may potentially prevent disease outbreaks such as Vibriosis, white feces disease, white-tale disease, and AHPND.

## Ethic Statement

This study did not sacrifice any organism, nor introduce viable pathogenic bacteria to the environment. All sampling procedures were already informed to and have been agreed upon by shrimp pond owners.

## Author Contributions

YA, JH, and AG designed the project. YA collected the samples and conducted the *in situ* measurement with the logistic support of AT and AK. YA and CH completed the statistical analysis. YA prepared the manuscript with input from all co-authors.

## Conflict of Interest Statement

The authors declare that the research was conducted in the absence of any commercial or financial relationships that could be construed as a potential conflict of interest.

## Footnotes

1 https://doi.pangaea.de/10.1594/PANGAEA.889316
