# Supplementary material for: Bacterial Abundance and Community Composition in Pond Water From Shrimp Aquaculture Systems With Different Stocking Densities
Source: Front Microbiol. 2018 Oct 18;9:2457. doi: 10.3389/fmicb.2018.02457 (PMC6200860; doi:10.3389/fmicb.2018.02457)
Supplement: Supplementary file 1 [file Table_1.DOCX]

Supplementary Material

Bacterial abundance and community composition in pond water from shrimp aquaculture system with different stocking densities

Yustian Rovi Alfiansah ^*^, Christiane Hassenrück, Andreas Kunzmann, Arief Taslihan, Jens Harder and Astrid Gärdes

*** Correspondence:** yustian.alfiansah@leibniz-zmt.de

# Supplementary Figures and Tables

# Supplementary Figures

# Supplementary Figure 1. Particle-attached bacterial cells from one of the water samples of intensive (A,C) and semi-intensive (B,D) ponds, at day 10 (A,B) and at day 40 (C,D). Bigger bright circles indicate plankton cells.

# Supplementary Figure 2. Rarefaction curves of OTU number and inverse Simpson index from 59 samples. Increasing color intensity indicates rearing time. Color yellow till dark red indicates samples from the intensive ponds, while grey to black indicates samples from the semi-intensive ponds.

# Supplementary Figure 3. Redundancy analysis (RDA) ordination showing bacterial community composition in relation to significant environmental variables. Point shape indicates replicate pond of the same system. Increasing color intensity indicates rearing time. Sal: Salinity, Chla: Chlorophyll a, NO_3_^-^: Nitrate.

## Supplementary Tables

**Supplementary Table 1.** Averages of bacterial cell concentrations (cell mL^-1^) and aggregate numbers (aggregates mL^-1^). Bacterial cell and aggregate numbers are presented in average ± standard deviation. Values without bracket indicate bacterial cell numbers. Values within bracket indicate aggregate numbers. Total bacterial cells were calculated by multiplication of the average of bacterial cell numbers, aggregates numbers, filter surface factor (x8) and then divided by filtered water (day 10: 10 ml, day 40,50 and 60: 1 ml, day 70: 0.5 ml).

| **Day** | **Aggregate sizes (µm²)** | | | | | | | | | |
| --- | --- | --- | --- | --- | --- | --- | --- | --- | --- | --- |
|  | **< 300** | | **300 - 625** | | **937.5 - 1,406.5** | | **1,725 - 2,500** | | **2,825 - 3,906.25** | |
|  | **S** | **T** | **S** | **T** | **S** | **T** | **S** | **T** | **S** | **T** |
| **10** | 607±62 | 526±58 | 57±12 (296±7) | 46±15 (338±23) | 86±3 (511±63) | 80±7 (398±13) | 100±13 (124±17) | 102±5 (111±11) | 160±24(24±5) | 195±14 (20±2) |
| **40** | 399±37 | 409±62 | 52±9 (246±41) | 56±2 (298±14) | 70±12 (494±27) | 72±6 (433±62) | 109±16 (182±10) | 112±23 (159±13) | 194±7 (90±4) | 202±12 (70±11) |
| **50** | 468±18 | 417±75 | 55±5 (273±19) | 44±9 (290±31) | 83±8 (345±25) | 82±4 (420±37) | 106±9 (201±10) | 99±3 (185±29) | 186±22(85±9) | 191±4 (84±8) |
| **60** | 403±44 | 435±41 | 53±4 (255±22) | 54±3 (251±8) | 74±9 (343±23) | 69±5 (353±8) | 96±3 (193±11) | 98±7 (198±14) | 190±12(99±8) | 192±27 (96±6) |
| **70** | 243±15 | 246±17 | 53±4 (142±14) | 54±3 (133±22) | 77±1 (275±13) | 79±2 (270±19) | 115±7 (85±7) | 99±5 (77±11) | 199±14(46±3) | 191±14 (52±10) |

S: semi-intensive system (3 ponds), T: Intensive system (3 ponds).
