# Supplementary material for: Bacterial Abundance and Community Composition in Pond Water From Shrimp Aquaculture Systems With Different Stocking Densities
Source: Front Microbiol. 2018 Oct 18;9:2457. doi: 10.3389/fmicb.2018.02457 (PMC6200860; doi:10.3389/fmicb.2018.02457)
Supplement: Supplementary file 2 [file Table_2.DOCX]

Supplementary Material

Bacterial abundance and community composition in pond water from shrimp aquaculture system with different stocking densities

Yustian Rovi Alfiansah ^*^, Christiane Hassenrück, Andreas Kunzmann, Arief Taslihan, Jens Harder and Astrid Gärdes

## Supplementary Table 2. Primer pairs for the toxin gene assay of *V. parahaemolyticus*

| **Primer sets^a^** | **Sequences** | **Length**  **(bp)** | **T_M_-value (°C)^b^** | **GC**  **(%)^c^** | **Amplicon size (bp)** | **Source** |
| --- | --- | --- | --- | --- | --- | --- |
| *toxR*_F | 5‘-CAG CGT TGT GAA GCA ACA TTA G-3‘ | 22 | 53 | 45.45 | 98 | Our study |
| *toxR*_R | 5‘-CTC CAG ATC GTG TGG TTG TAT G-3‘ | 22 | 54.8 | 50.0 |  |  |
|  |  |  |  |  |  |  |
| *tlh*_F | 5‘-CCGTCAGATTGGTGAGTATCAG-3‘ | 22 | 54.8 | 50.0 | 99 | Our study |
| *tlh*_R | 5‘-CGTTCAATGCACTGCTCAATAG-3‘ | 22 | 53 | 45.45 |  |  |
|  |  |  |  |  |  |  |
| *tdh*_F | 5’-CAGTATTCACAACGTCWGGTACTA-3’ | 24 | 54 | 41.67 | 200 | Our study |
| *tdh*_R | 5’-TGGAATAGAAYCTTCATCTTCACC-3’ | 24 | 54 | 37.5 |  |  |
|  |  |  |  |  |  |  |
| *PirA*_F | 5’-GTCGGTCGTAGTGTAGACATTG-3’ | 22 | 57.4 | 50 | 147 | Our study |
| *PirA*_R | 5’-AGGGCGTTGTAAATGGTAAGT-3’ | 21 | 57 | 42.9 |  |  |
|  |  |  |  |  |  |  |
| *PirB*_F | 5’-GGTGATGAATGGCTTGGTTATG-3’ | 22 | 56.9 | 45.5 | 124 | Our study |
| *PirB*_R | 5’-GCACATCAGAATCGGTGAAAC-3’ | 21 | 56.8 | 47.6 |  |  |

^a^*tlh*: thermolabile hemolysin, *tdh*: thermostable direct hemolysin, *pirA* and *PirB*: Photorhabdus insect-related (Pir) toxins

^b^ T_M_: Melting temperature

^c^ GC: Guanine-cytosine content
