# Supplementary material for: Bacterial Abundance and Community Composition in Pond Water From Shrimp Aquaculture Systems With Different Stocking Densities
Source: Front Microbiol. 2018 Oct 18;9:2457. doi: 10.3389/fmicb.2018.02457 (PMC6200860; doi:10.3389/fmicb.2018.02457)
Supplement: Supplementary file 3 [file Table_3.DOCX]

Supplementary Material

Bacterial abundance and community composition in pond water from shrimp aquaculture system with different stocking densities

Yustian Rovi Alfiansah ^*^, Christiane Hassenrück, Andreas Kunzmann, Arief Taslihan, Jens Harder and Astrid Gärdes

**Supplementary Table 3**. List of samples tested for toxin genes of *V. parahaemolyticus* (*toxR*, *tlh*, *tdh, pirAB*)

| Number | Fraction | Pond | Day | System |
| --- | --- | --- | --- | --- |
| 1 | FL | T1 | 10 | Intensive |
| 2 | PA | T1 | 10 | Intensive |
| 3 | FL | S1 | 10 | Semi-intensive |
| 4 | PA | T2 | 40 | Intensive |
| 5 | FL | T2 | 50 | Intensive |
| 6 | PA | T2 | 40 | Intensive |
| 7 | PA | T3 | 10 | Intensive |
| 8 | FL | S1 | 60 | Semi-intensive |
| 9 | FL | S2 | 50 | Semi-intensive |
| 10 | FL | S2 | 40 | Semi-intensive |
