# Supplementary material for: Bacterial Abundance and Community Composition in Pond Water From Shrimp Aquaculture Systems With Different Stocking Densities
Source: Front Microbiol. 2018 Oct 18;9:2457. doi: 10.3389/fmicb.2018.02457 (PMC6200860; doi:10.3389/fmicb.2018.02457)
Supplement: Supplementary file 4 [file Table_4.DOCX]

Supplementary Material

Bacterial abundance and community composition in pond water from shrimp aquaculture system with different stocking densities

Yustian Rovi Alfiansah ^*^, Christiane Hassenrück, Andreas Kunzmann, Arief Taslihan, Jens Harder and Astrid Gärdes

**Supplementary Table 4**. General Linear Mix Models (GLMM) for physical parameters, the abundances of cultivable heterotrophic bacteria and inorganic nutrient concentrations.

| Parameters^a^ | System | | | | Day | | | | Interaction between system and day | | | |
| --- | --- | --- | --- | --- | --- | --- | --- | --- | --- | --- | --- | --- |
|  | dfn^b^ | dfd^c^ | F-value | p-value | dfn^b^ | dfd^c^ | F-value | p-value | dfn^b^ | dfd^c^ | F-value | p-value |
| Temperature | 1 | 4 | 2.425 | 0.194 | 6 | 24 | 7.252 | <0.001 | 6 | 24 | 5.706 | 0.001 |
| Salinity | 1 | 4 | 8.485 | 0.044 | 6 | 24 | 6.471 | <0.001 | 6 | 24 | 3.086 | 0.022 |
| pH | 1 | 4 | 12.446 | 0.024 | 6 | 24 | 3.479 | 0.013 | 6 | 24 | 1.256 | 0.314 |
| DO | 1 | 4 | 10.501 | 0.032 | 6 | 24 | 1.814 | 0.139 | 6 | 24 | 0.885 | 0.521 |
| Turbidity | 1 | 4 | 1.258 | 0.325 | 6 | 24 | 7.885 | <0.001 | 6 | 24 | 2.775 | 0.034 |
| Chl a | 1 | 4 | 7.315 | 0.054 | 6 | 24 | 6.228 | <0.001 | 6 | 24 | 3.809 | 0.008 |
| Phosphate | 1 | 4 | 0.035 | 0.86 | 6 | 24 | 1.033 | 0.428 | 6 | 24 | 1.079 | 0.402 |
| Nitrite | 1 | 4 | 0.437 | 0.545 | 6 | 24 | 0.493 | 0.807 | 6 | 24 | 2.673 | 0.039 |
| Nitrate | 1 | 4 | 0.648 | 0.466 | 6 | 24 | 0.907 | 0.507 | 6 | 24 | 0.226 | 0.964 |
| Ammonium | 1 | 4 | 0.196 | 0.681 | 6 | 24 | 0.767 | 0.603 | 6 | 24 | 0.687 | 0.662 |
| Silicate | 1 | 4 | 2.858 | 0.166 | 6 | 24 | 0.802 | 0.578 | 6 | 24 | 1.163 | 0.358 |
| SPM | 1 | 4 | 13.197 | 0.022 | 6 | 24 | 121.978 | <0.001 | 6 | 24 | 1.538 | 0.209 |
| THB | 1 | 4 | 0.99 | 0.376 | 6 | 24 | 102.191 | <0.001 | 6 | 24 | 6.519 | <0.001 |
| TPPV | 1 | 4 | 0.11 | 0.757 | 6 | 24 | 15.917 | <0.001 | 6 | 24 | 2.694 | 0.038 |

^a^Chla: Chlorophyll a, SPM:Suspended particulate matter, THB: Total heterotrophic bacteria, TPPV: Total cultivable potential pathogenic *Vibrio*,

^b^dfn: degrees of freedom numerator,

^c^dfd: degrees of freedom denominator
