# Supplementary material for: Bacterial Abundance and Community Composition in Pond Water From Shrimp Aquaculture Systems With Different Stocking Densities
Source: Front Microbiol. 2018 Oct 18;9:2457. doi: 10.3389/fmicb.2018.02457 (PMC6200860; doi:10.3389/fmicb.2018.02457)
Supplement: Supplementary file 5 [file Table_5.docx]

Supplementary Material

Bacterial abundance and community composition in pond water from shrimp aquaculture system with different stocking densities

Yustian Rovi Alfiansah ^*^, Christiane Hassenrück, Andreas Kunzmann, Arief Taslihan, Jens Harder and Astrid Gärdes

**Supplementary Table 5**. Multivariate analysis of variance (MANOVA) for bacterial cell numbers from DAPI counting

| Variables | Df | Pillai | Approx. F | Dfn | Dfd | Pr(>F) |
| --- | --- | --- | --- | --- | --- | --- |
| Day | 4 | 1.515 | 12.48 | 8 | 32 | < 0.001 |
| System | 1 | 0.264 | 0.54 | 2 | 3 | 0.63 |
| Interaction | 4 | 0.274 | 0.64 | 8 | 32 | 0.74 |
| Residual | 16 |  |  |  |  |  |

Df: degrees of freedom, dfn: degrees of freedom numerator, dfd: degrees of freedom denominator
