# Supplementary material for: Bacterial Abundance and Community Composition in Pond Water From Shrimp Aquaculture Systems With Different Stocking Densities
Source: Front Microbiol. 2018 Oct 18;9:2457. doi: 10.3389/fmicb.2018.02457 (PMC6200860; doi:10.3389/fmicb.2018.02457)
Supplement: Supplementary file 6 [file Table_6.docx]

Supplementary Material

Bacterial abundance and community composition in pond water from shrimp aquaculture system with different stocking densities

Yustian Rovi Alfiansah ^*^, Christiane Hassenrück, Andreas Kunzmann, Arief Taslihan, Jens Harder and Astrid Gärdes

**Supplementary Table 6**. General Linear Mixed Models (GLMM) for significant predictor variable (Day)

| Parameters^a^ | Day | | | |
| --- | --- | --- | --- | --- |
|  | Dfn | Dfd | F-value | p-value |
| PA cells | 4 | 16 | 260.283 | <0.001 |
| FL cells | 4 | 16 | 15.940 | <0.001 |
