# Supplementary material for: Bacterial Abundance and Community Composition in Pond Water From Shrimp Aquaculture Systems With Different Stocking Densities
Source: Front Microbiol. 2018 Oct 18;9:2457. doi: 10.3389/fmicb.2018.02457 (PMC6200860; doi:10.3389/fmicb.2018.02457)
Supplement: Supplementary file 7 [file Table_7.docx]

Supplementary Material

Bacterial abundance and community composition in pond water from shrimp aquaculture system with different stocking densities

Yustian Rovi Alfiansah ^*^, Christiane Hassenrück, Andreas Kunzmann, Arief Taslihan, Jens Harder and Astrid Gärdes

**Supplementary Table 7.** R value of the post-hoc of analysis of similarity (ANOSIM) for bacterial community composition

| Intensive | | | | | | Semi-intensive | | | |
| --- | --- | --- | --- | --- | --- | --- | --- | --- | --- |
| Fractions | Day | 10 | 40 | 50 | 60 | 10 | 40 | 50 | 60 |
| FL | 10 |  |  |  |  |  |  |  |  |
|  | 40 | 0.22 |  |  |  | -0.15 |  |  |  |
|  | 50 | -0.11 | -0.22 |  |  | -0.37 | 0.04 |  |  |
|  | 60 | 0.11 | 0.26 | 0.15 |  | -0.19 | -0.04 | -0.22 |  |
|  | 70 | 0.44 | 0.67 | 0.11 | 0.59 | -0.04 | 0.07 | -0.22 | -0.26 |
| PA | 10 |  |  |  |  |  |  |  |  |
|  | 40 | -0.04 |  |  |  | -0.22 |  |  |  |
|  | 50 | -0.04 | -0.15 |  |  | -0.07 | -0.19 |  |  |
|  | 60 | 0.44 | -0.19 | 0.19 |  | 0.00 | 0.25 | -0.25 |  |
|  | 70 | 0.56 | -0.04 | 0.11 | -0.41 | -0.11 | -0.07 | -0.07 | -0.75 |

FL: Free-living, PA: particle-attached
