# Supplementary material for: Bacterial Abundance and Community Composition in Pond Water From Shrimp Aquaculture Systems With Different Stocking Densities
Source: Front Microbiol. 2018 Oct 18;9:2457. doi: 10.3389/fmicb.2018.02457 (PMC6200860; doi:10.3389/fmicb.2018.02457)
Supplement: Supplementary file 8 [file Table_8.docx]

Supplementary Material

Bacterial abundance and community composition in pond water from shrimp aquaculture system with different stocking densities

Yustian Rovi Alfiansah ^*^, Christiane Hassenrück, Andreas Kunzmann, Arief Taslihan, Jens Harder and Astrid Gärdes

**Supplementary Table 8. Correlation between *Vibrio* FRAxC and selected biogeochemical parameters in free-living (FL) and particle-attached (PA) fractions**

|  | *Spearman* correlation coefficients | |
| --- | --- | --- |
| Parameters | FL | PA |
| SPM | 0.38 | 0.21 |
| Temperature | 0.26 | 0.03 |
| pH | -0.29 | -0.36 |
| Salinity | -0.28 | -0.14 |
| Ammonium | 0.27 | 0.17 |
| Nitrite | -0.18 | 0.14 |
| Phosphate | -0.12 | 0.11 |
| NP^a^ ratio | 0.18 | 0.13 |
| TPPV^b^ | 0.27 | 0.24 |

^a^ NP: Nitrogen-Phosphate

^b^ TPPV: Total cultivable potential pathogenic *Vibrio*
